# Improved specificity for detection of *Mycobacterium bovis *in fresh tissues using IS*6110 *real-time PCR

**DOI:** 10.1186/1746-6148-7-50

**Published:** 2011-08-25

**Authors:** Tyler C Thacker, Beth Harris, Mitchell V Palmer, Wade R Waters

**Affiliations:** 1 Infectious Bacterial Diseases Research Unit, National Animal Disease Center, Agricultural Research Service, United States Department of Agriculture, Ames, Iowa, USA; 2 Mycobacteria and Brucella Section, National Veterinary Services Laboratories, United States Department of Agriculture, Ames, Iowa, USA

## Abstract

**Background:**

Culture of *M. bovis *from diagnostic specimens is the gold standard for bovine tuberculosis diagnostics in the USA. Detection of *M. bovis *by PCR in tissue homogenates may provide a simple rapid method to complement bacterial culture. A significant impediment to PCR based assays on tissue homogenates is specificity since mycobacteria other than *M. bovis *may be associated with the tissues.

**Results:**

Previously published IS*6110 *based PCR diagnostic assays, along with one developed in house, were tested against environmental mycobacteria commonly isolated from diagnostic tissues submitted to the National Veterinary Services Laboratory. A real-time PCR assay was developed (IS6110_T) that had increased specificity over other IS*6110 *based assays. Of the 13 non-tuberculous mycobacteria tested with IS6110_T only *M. wolinskyi *was positive. Thirty *M. bovis *infected tissue homogenates and 18 control tissues were used to evaluate the potential for the assay as a diagnostic test. In this small sample, IS6110_T detected 20/30 samples from *M. bovis *infected animals and 0/18 control tissues.

**Conclusions:**

The IS6110_T assay provides a PCR based assay system that is compatible with current diagnostic protocols for the detection of *M. bovis *in the USA and compliments current testing strategies.

## Background

*Mycobacterium bovis *is the causative agent of bovine tuberculosis. Isolation of *M. bovis *from tissues harvested from suspect animals remains the gold standard for diagnosis. Although bacteriological culture is a reliable, definitive method for detection of *M. bovis *it takes a significant amount of time. Detecting the presence of *M. bovis *DNA in tissues prepared for culture may provide a simple, rapid diagnostic method.

Mycobacteria other than *M. bovis *are routinely isolated from tissues submitted for diagnostic culture. These non-*M. bovis *mycobacteria may interfere with PCR diagnostic tests performed on these tissues if the test does not have sufficient specificity. Most PCR primer pairs published to date were designed for identification of mycobacteria that have been isolated by culture. Use of selective media and biochemical tests can eliminate many non-*M. bovis *mycobacteria prior to PCR tests; however, PCR assays on tissues that may have non-*M. bovis *mycobacteria requires sufficient specificity to differentiate environmental mycobacteria from *M. bovis*.

To address this possibility, DNA from strains of mycobacteria other than *M. bovis*, that are routinely isolated from diagnostic samples, were tested for cross reactivity with the commonly used IS*6110 *PCR primer pairs used to detect *M. bovis*.

## Results

### Specificity of PCR primers

The following mycobacteria are routinely isolated from tissues submitted for *M. bovis *diagnostic culture at the National Veterinary Services Laboratories: *M. smegmatis, M. terrae, M. goodii, M. fortuitum, M. kansasii, M. wolinskyi, M. simiae, M. peregrinum, M. intracellulare, M. chelonae, M. avium *subsp *avium*, and *M.avium *subsp *paratuberculosis*. DNA from these strains were used to determine the specificity of two previously published PCR primer pairs IS41/43[1] and IS6110[2]. Both sets of primers generated a PCR product for *M. bovis, M. terrae, M. goodii*, and *M. wolinskyi *(Table 1). The IS41/43 primer pair also detected *M. fortuitum*; whereas, IS6110 primer pair cross-reacted with *M. peregrinum *and *M. chelonae*.

**Table 1:** Comparison of primer specificity using reference strains of Mycobacteria commonly isolated from clinical samples.

	Primers			
	
	Conventional PCR			Real-time PCR
**Mycobacterium species**	**IS6110^a^**	**IS41/IS43^b^**	**IS6110_T**	**IS6110_T**

*M. bovis*	+	+	+	+
*M. terrae*	+	+		
*M. goodii*	+	+	+	
*M. fortuitum*		+		
*M. kansasii*				
*M. avium *subsp. *Paratuberculosis*				
*M. wolinskyi*	+	+	+	+
*M. simiae*				
*M. peregrinum*	+			
*M. intracellulare*				
*M. chelonae*	+			
*M. avium *subsp *avium*				
*M. smegmatis*				

^a ^[2]

^b ^[1]

### Development of real-time PCR primers with increased specificity

The cross reactivity of previously published primers with commonly isolated environmental mycobacteria necessitated the development of primers with greater specificity. Primers and probe for real-time PCR were developed that targeted IS*6110 *(IS6110_T). The primers were tested for specificity using the mycobacteria described above (Table 1). The IS6110_T primer pair generated a PCR product from *M. bovis, M. wolinskyi *and *M. goodii *using conventional PCR; however, using the primers and probe in a real-time PCR assay resulted in a positive reaction to only *M. bovis *and *M. wolinskyi *(Table 1). A 317 bp region of *M. wolinskyi *and *M. bovis *genomes that contained the region amplified by the primers used in this paper were sequenced to determine if a test with higher specificity could be developed using this region. The amplified region from *M. wolinskyi *was 100% identical to that of *M. bovis*. Portions of the 16S gene was sequenced to confirmed the identity of *M. wolinskyi*.

### Establishing assay conditions

The limit of detection for IS6110_T was 100 fg of *M. bovis *DNA. The limit of detection was determined using serial dilutions of *M. bovis *DNA. Clinical tissue samples will contain bovine (or other species being tested) DNA that may inhibit the real-time PCR reaction. To determine the effect of non-target DNA on the real-time PCR reaction, increasing amounts of bovine DNA was spiked with *M. bovis *DNA. *M. bovis *was detectable in up to 1 μg of bovine DNA. At 2 μg of bovine DNA inhibition of the PCR was detected.

To test the utility of this assay to detect *M. bovis *in clinical samples, DNA was isolated from tissue homogenates that were culture positive for *M. bovis *or *M. smegmatis*. Real-time PCR using primers for *M. bovis *and bovine β-actin was performed on each sample in duplicate. β-actin served as a control to detect inhibition of the PCR reaction. A sample was considered positive when a PCR product was detected in both duplicates from β-actin and IS*6110*. Initial studies using both conventional and real-time PCR assays only detected 2/10 and 1/10 samples respectively. It was hypothesized that the low level of detection was due to relatively little *M. bovis *DNA relative to bovine DNA present in samples. To test this hypothesis, DNA from 4 homogenized tissues that were culture positive for *M. bovis *and one that was culture positive for *M. smegmatis *were chosen. Real-time PCR reactions were prepared with increasing amounts of DNA from these samples (100 ng to 1000 ng). One of the four *M. bovis *culture positive samples was PCR positive when 100 ng and two of four when 250 ng of DNA was used in the PCR reaction (Table 2). Increasing DNA concentration did not result in detection of the remaining samples.

**Table 2:** Results of increasing starting DNA on real-time PCR assay performance.

	Sample									
	01916^a^		4022^a^		4477^b^		01870^a^		04034^a^	
**DNA^c^**	**IS6110_T**	**β-actin**	**IS6110_T**	**β-actin**	**IS6110_T**	**β-actin**	**IS6110_T**	**β-actin**	**IS6110_T**	**β-actin**

100	+	+	-	+	-	+	-	+	-	+
250	+	+	+	+	-	+	-	+	-	+
500	+	+	+	+	-	+	-	+	-	+
1000	+	+	+	+	-	+	-	+	-	+

^a ^*M. bovis *infected tissues

^b ^*M. smegmatis *infected tissues

^c ^DNA isolated from homogenized tissues added to PCR reaction in nanograms.

To increase the sensitivity of the assay, a nested PCR strategy was developed. Initially, conventional PCR was performed using primer IS41 and the IS6110_T reverse primer to generate a 334 base pair product that encompasses the region targeted by the real-time PCR assay. Five microliters of the initial PCR reaction was used as the template in the real-time PCR assay. The samples described above were tested using the nested assay. *M. bovis *DNA was detected in all four of the samples from infected tissues.

### Detection of *M. bovis *in tissues from naturally infected animals

Additional tissue homogenates were obtained to test the diagnostic potential of this assay. DNA was isolated from tissues of *M. bovis *infected cattle (n = 30) and non-*M. bovis *infected animals (n = 18). Of the 30 *M. bovis *culture positive tissues 20 were PCR positive; whereas, no PCR product was detected in the non-*M. bovis *infected tissues (Table 3).

**Table 3:** Results of real-time PCR using IS6110_T to detect *M. bovis *in diagnostic samples.

	Culture Results	
**Result from PCR assay**	***M. bovis***	**Non-*M. bovis***

Positive	20	0
Negative	10	18

## Discussion

A number of factors may affect the ability of a PCR based test to be successful. Uncharacterized PCR inhibitors can be carried over from DNA isolation procedures from blood and tissues. Although these may be a cause of concern, they are irrelevant if primers and probes with sufficient specificity are not available. Environmental mycobacteria present in lymph nodes submitted for diagnostic testing can confound assays that lack sufficient specificity. Detection of *M. tuberculosis *complex species using primers to IS*6110 *is well established; as evidenced by the large number of publications referencing the original publication by Eisenach *et al*. [2]. In the USA, IS*6110 *is the diagnostic target used at National Veterinary Services Laboratories for detection of *M. bovis *in fixed tissues [3,4]. Most publications testing the specificity of the method of Eisenach *et al*. are designed to detect *M. tuberculosis *with specificity being tested against mycobacteria thought to be common in humans. Because cattle may be infected with a different subset of non-tuberculous mycobacteria than those infecting humans, we sought to determine if established PCR protocols targeting IS*6110 *would be appropriate for strains isolated from cattle in the USA. In the present study, Eisenach *et al*. primers cross-reacted with mycobacteria other than *M. tuberculosis *complex species (Table 1). Redesign of the primers and the addition of a probe increased the specificity against strains commonly isolated from cattle in the USA.

Cross-reactivity to *M. wolinskyi *remained after redesign of the probe used in the real-time PCR reaction. Sequencing of the region targeted by the IS*6110 *primers and probes revealed that the region was identical between *M. bovis *and *M. wolinskyi*. The region of the IS*6110 *that is targeted by these primers is in the 5' region of the transposase gene, which may be common between IS*6110 *and another insertion sequence in *M. wolinskyi*. The 16S gene of the *M. wolinskyi *sample used in this study was sequenced to confirm the identity of the isolate. The impact of this cross reactivity will be relatively minor since *M. wolinskyi *was only isolated 3 times between 2004 and October 2010.

Thirty *M. bovis *infected and 18 non-*M. bovis *infected tissues were used to test the utility of this assay. None of the tissues from non-*M. bovis *infected animals were positive by PCR. Although the sample size is small the apparent specificity is 100% (95% CI, 44.1%-89.5%).

This assay detected *M. bovis *DNA in 20 of the 30 culture positive samples tested, with an apparent sensitivity of 66.7% (95% CI, 47.2%-82.7%). Several methodologies have previously been employed to increase sensitivity. Parra *et al*. used a capture probe to isolate mycobacterial DNA from tissue homogenates [5] achieving a similar sensitivity (65.6%) to that reported here. Taylor *et al*. reported a sensitivity of 70% when performing PCR directly on tissue homogenates, but increased the sensitivity to 91% when PCR was only performed on DNA isolated from lesions excised from the tissues rather than whole tissue homogenates [6]. The limitation of this method is that the assay can only be performed on tissues that have visible lesions, thus excluding samples without readily apparent lesions.

## Conclusions

The use of a PCR assay to detect *M. bovis *in tissue homogenates may provide a more rapid method for providing diagnostic test results to field veterinarians than culture. The IS6110_T real-time PCR assay provides increased specificity over previously published IS*6110 *assays with a sensitivity of 66.7%. A large-scale study is needed to determine if the sensitivity of the assays are adequate for the Bovine TB Eradication Program in the USA.

## Methods

### Mycobacterial Strains

The following reference strains obtained from ATCC: *M. smegmatis *(ATCC 35797), *M. terrae *(ATCC 15755), *M. goodii *(ATCC 700504), *M. fortuitum *(ATCC 6841), *M. kansasii *(ATCC 12478), *M. wolinskyi *(ATCC 700010), *M. simiae *(ATCC 25275), *M. peregrinum *(ATCC 14467), *M. intracellulare *(ATCC 13950) and *M. chelonae *(ATCC 35752). *M. bovis, M. avium*, and *M. avium *subspecies *paratuberculosis *were well characterized clinical field isolates. Mycobacteria were grown in grown in Middlebrook 7H9 medium supplemented with 10% oleic acid-albumin-dextrose complex (Becton Dickinson Microbiology Systems, Franklin Lakes, NJ. Mycobactin J was added to culture media when growing *M. paratuberculosis*. DNA was isolated using the following method. Mycobacteria from 1.5 ml o f culture were washed once with 1 × Tris-EDTA (TE) buffer and resuspended in 500 μl TE. Twenty microliters of 100 mg/ml Lysozyme (Sigma, St. Louis, MO) was added and incubated for a minimum of 3 hours or overnight at 37°C. SDS was added to a final concentration of 1% followed by the addition of 150 μg of Proteinase K (Roche Applied Science, Indianapolis, IN) and incubated for 3 hours at 65°C. Following protein digestion, 100 μl of 5 M NaCl was added. One hundred microliters of CTAB/NaCl (274 mM hexadecyltrimethylammonium bromide/0.7 M NaCl, Sigma). The resulting solution was mixed thoroughly by inverting tube then incubated at 65°C for 10 minutes. An equal volume of Phenol/chloroform/isoamyl alcohol (25:24:1) was added and an emulsion formed by vortexing then centrifuged at 16,000 × g for 5 minutes at room temperature.

The aqueous phase was removed to a fresh tube and equal volume of isopropanol was added and the samples were placed at -80°C. Precipitated DNA was collected by centrifugation at 16,000 × g for 150 minutes at 4°C. The isopropanol was removed and 1 ml of ice-cold 70% ethanol was added. The sample was centrifuged at 16,000 × g for 5 minutes and the ethanol removed. The samples were then dried at room temperature until dry. The DNA pellet was resuspended in PCR grade water.

### Isolation of DNA from fresh tissues

Forty-eight (42 bovine, 5 cervine, 1 porcine) homogenized lymph nodes submitted as part of USDA's National Bovine Tuberculosis Eradication Program were obtained from the Mycobacteria and Brucella Section at the National Veterinary Services Laboratories during 2005 and 2006. Homogenization and decontamination of tissues was performed according to established protocols [7] in preparation for recovery of *M. bovis *by culture. Homogenates not used to inoculate culture media were frozen at -80°C until used in PCR assays. Five hundred microliters of the homogenate was heat inactivated by incubating the sample in a screw-topped microcentrifuge tube at 80°C for 40 minutes. After 20 minutes the sample was mixed. Particulate matter (including mycobacteria) was collected by centrifuging the sample for 10 minutes at 13,000 × g. The supernatant was discarded and the pellet resuspended in 500 μl ASL buffer (Qiagen, Valencia, CA) followed by a 5 min incubation at 95°C. After cooling to 56°C, 125 μg of Proteinase K (Sigma, St. Louis, MO) was added to samples. Buffer AL (Qiagen) was then added and the sample was incubated overnight at 56°C. Liberated DNA was isolated using the Qiamp Blood Mini Kit according to the manufacturer's directions except that 500 μl of cold 100% ethanol was added before the sample was applied to the column.

### Detection of mycobacterial DNA by conventional PCR

The following primer pairs were tested IS41/43 [1], IS6110 [2], and primers designed in house IS6110_T forward (5'-AGTTTGGTCATCAGCCGTTC-3') and IS6110_T reverse (5'-CGAACTCAAGGAGCACATCA-3') using Primer3 [8]. The following optimized protocols were used with 50 μl PCR reactions.

IS41/43: A 50 μl reaction was prepared using 1 × PCR reaction buffer (final MgCl_2 _2 mM), 1 U FastStart Taq, 2.5 mM each dNTP's (Roche), 5 μg BSA (Ambion), 2.5 pmol of each primer, and with 0.5 μl DNA. A touch down PCR method was used to reduce false priming and increase specificity. The following conditions were used: an initial activation step of 94°C for 2 min. The following cycle was repeated 7 times: 94°C for 45 seconds, 72°C for 1 min (-1°C/cycle), then 72°C extension for 2 min. Amplification was continued by cycling through the following conditions for an additional 28 times: 94°C for 1 minute, 65°C for 1 min then 72°C for 2 min. A final 10 min extension was performed.

IS6110_T: 50 μl reactions were prepared as for the IS41/43 primers except that 1 pmol of each primer was added. PCR with these primers was performed using the following thermocycler conditions: an initial activation step of 94°C for 2 min. The following cycle was repeated 14 times: 94°C for 45 seconds, 65°C for 1 min (-1°C/cycle), then 72°C extension for 2 min. Amplification was continued by cycling through the following conditions an additional 28 times: 94°C for 1 minute, 50°C for 1 min then 72°C for 2 min. A final 10 min extension was performed.

IS6110 Primers: A 50 μl reaction was prepared with the following components: 1 × Gene Amp PCR Buffer II (final MgCl_2 _concentration 2.5 mM), 1.25 U AmpliTaq Gold (Applied Biosystems), 2.5 mM each dNTP (Roche), 2.5 pmol each primer, and 0.5 μl template.

All PCR products were analyzed on a 1.5% agarose gel (Invitrogen) stained with ethidium bromide and visualized with a Bio-Rad Gel Doc XR.

### Detection of mycobacterial DNA by real-time PCR

Detection of Mycobacterial DNA by real-time PCR was performed using the IS6110_T primers and a 5' Hex labeled probe (5'-AGCCACACTTTGCGGGCACC-3') with a 3' Iowa Black FQ quencher (Integrated DNA Technologies). Taqman Universal PCR Mastermix (Applied Biosystems) was used according to the manufacturer's directions with a final primer concentration of 0.4 μM each and 0.1 μM probe. The real-time PCR was run in an ABI7500. Detection of β-actin was used as a PCR control.

### Detection of β-actin

PCR reactions to detect mammalian β-actin were performed parallel with each sample. The primers 5'- TCCCTGGAGAAGAGCTACGA -3' and 5'- AGGAAGGAAGGCTGGAAGAG -3' and FAM labeled probe 5'- TCACCATCGGCAATGAGCGG -3' with a 3' Iowa Black FQ quencher (Integrated DNA Technologies) were designed using Primer3Plus [9]. β-actin PCR was performed as described for IS6110_T. β-actin was included as a positive control for the PCR reaction and to detect successful DNA extraction from tissue homogenates. Lack of amplification was assumed to indicate that the PCR reaction was inhibited. For a sample to be considered positive PCR products from both the IS*6110 *PCR and β-actin PCR needed to occur.

### Nested real-time PCR

The initial outside PCR reaction was carried out as described for IS6110_T (conventional PCR) except 2.5 pmol of the primers IS41 and IS6110_T reverse with 5 μg bovine serum albumin (Ambion). The second step was carried out as described above.

### Sequencing

IS*6110 *Products: PCR products were excised from a 1% agarose gel after electrophoresis. The PCR products were extracted from the gel and purified using a Gel extraction Kit (Qiagen) according to the manufacturer's instructions. Sequencing was performed by the Genomics facility at the National Animal Disease Center using standard techniques.

16S Gene: Primers T39 and T13 from Talaat *et al*. [10] were used to PCR amplify a portion of the 16S Gene. The PCR product was purified using a MinElute PCR Purification Kit (Qiagen) according to the manufacturer's instructions. The purified product was sequenced as above. The sequence was identified as *M. wolinskyi *using the Ridom Databse with 100% identity [11].

### Statistical analysis

Calculation of the diagnostic specificity and sensitivity along with 95% confidence intervals (CI) were done using epiR package (version 0.9-26) in R (version 2.11.1) [12].

## Authors' contributions

TCT conceived the study, participated in its design, data acquisition and analysis, and wrote the paper. BH participated in design of the study and data analysis. MVP and WRW participated in study design and data interpretation. All authors read and approved the final manuscript.
